# Effects of diets with different amino acid release characteristics on the gut microbiota and barrier function of weaned pigs

**DOI:** 10.1186/s12866-023-02762-8

**Published:** 2023-01-19

**Authors:** Mengmeng Mi, Zhiwen Shen, Nianzhi Hu, Qiyu Zhang, Bin Wang, Li Pan, Guixin Qin, Nan Bao, Yuan Zhao

**Affiliations:** Key Laboratory of Animal Production, Product Quality and Security, Ministry of Education, Jilin Provincial Key Laboratory of Animal Nutrition and Feed Science, College of Animal Science and Technology, Jilin Agricultural University, Jilin Agricultural University, Changchun, 130118 China

**Keywords:** Weaned pigs, Amino acid release, Intestinal barrier function, Gut microbiota

## Abstract

**Background:**

The absorption and utilization of proteins by animals is affected by the amino acid (AA) release characteristics of their diets. In the present study, we aimed to determine the effects of diets with various amino acid release characteristics on the intestinal barrier function and diversity of gut microbiota of weaned pigs.

**Results:**

Forty-eight pigs (7.45 ± 0.58 kg) were fed with diets having different amino acid release characteristics during a period of 28 days. We used a 2 × 3 full-factor (two protein levels and three protein sources with differing amino acid release characteristics) experimental design, with normal (standard terminal ileal digestibility of 17.5%) or low (standard terminal ileal digestibility of 14.9%) protein levels as the first factor. Casein (CAS), corn gluten meal (CGM) and a MIX diet were used as protein sources. Due to the more balanced release of amino acids, the diamine oxidase (DAO) concentrations in the CAS and MIX groups were significantly lower than those in the CGM group (*P* < 0.05); Reducing the dietary protein content from 17.5% to 14.9% had no significant effects on the levels of serum DAO or D-lactic acid. By contrast, it increased the microbial diversity (chao1 and ACE values) and the number of Lactobacillus in the jejunum (*P* < 0.05). The CAS-containing diet and the MIX diet resulted in significantly higher microbial diversity (Simpson and Shannon) than the CGM-containing diet in the jejunum.

**Conclusion:**

The balanced release of amino acids in CAS and MIX diets maintained intestinal barrier function and increased gut microbiota diversity. These findings could potentially provide a scientific reference for the rational preparation of piglet feed.

**Supplementary Information:**

The online version contains supplementary material available at 10.1186/s12866-023-02762-8.

## Introduction

According to the traditional theory of pig amino acid nutrition, diets formulated according to the ideal amino acid pattern that is consistent with the composition and proportion of amino acids and the pig body composition can result in optimal growth performance [1]. However, our previous studies have shown that although the protein content and digestible amino acids in terminal ileum of diets formulated with different protein sources are the same, there are significant differences in in vitro amino acid release patterns, and the in vivo nitrogen deposition rate is still not ideal [2, 3]. This is due to the decomposition of amino acids in the intestinal tract, which leads to the low utilization of dietary amino acids in pigs [4].

Small peptides and hydrolytic acids free from dietary decomposition in the gut can serve as the main ammonia source and fermentation substrate for intestinal microorganisms, but the flora involved in protein and amino acid metabolism is different in different intestinal segments [5]. High-protein diets and poor protein digestibility increase the production of potential pathogenic bacteria and harmful metabolites during intestinal protein fermentation [6, 7]. In addition, recent studies have shown that metabolites produced by intestinal microorganisms can activate host aromatic hydrocarbon receptors to regulate intestinal immune function and homeostasis [8–11]. These metabolites can also regulate intestinal microbial diversity and composition to maintain tryptophan metabolic balance in piglets [12]. Therefore, differences in characteristics of amino acid release kinetics may affect the ability of intestinal barrier to absorb and utilize nutrients, thus affecting physiological function and health status of the body. Thus, feeding pigs with low and high quality protein are two effective strategies for reducing protein fermentation in human and animals.

Based on previous studies, the same terminal ileum digestible diets supplemented with casein result in better synchronicity of amino acid release than those supplemented with corn gluten meal. In addition, diets with good synchronicity of amino acid release can improve nitrogen use efficiency in pigs [2, 3, 13]. Therefore, we selected a traditional corn-soybean meal diet supplemented with CAS, CGM or a mixture of both (CAS + CGM; MIX), to create diets with three different amino acid release characteristics, with the aim of further exploring the effects of different amino acid release rates on intestinal barrier function and intestinal microflora of piglets.

## Materials and Methods

### Animals

Forty-eight Duroc × Landrace × Yorkshire barrows were weaned at 21 days of age, at a mean body mass of 7.45 ± 0.58 kg. The piglets were then randomly allocated to six groups of eight and housed in separate pens at Jilin Agricultural University. The study was performed in the livestock houses of Jinlin Agricultural University which is maintained at a temperature of 25℃. The piglets were fed three times per day, at 7:00 am, 12:00 am, and 6:00 pm each day. Healthy weaned piglets with a similar perinatal period, similar parity, and body weight of about 7 kg under the same environment were selected for sampling. We used a 2 × 3 full-factor (two protein levels and three protein sources with different amino acid release characteristics) design for the study.

### Diets

The ingredients and nutrient contents of the diets were the same as our previous study of [2] Hu et al. (2022) and are shown in Table 1. The diets were formulated to meet NRC (2012) requirements, according to which the protein content of a diet can only be reduced by 2–4% by replacing protein content with monomeric amino acids, so that animal health is maintained and there are few adverse effects on nutrition, such as nitrogen deposition. The piglets were assigned to six groups: those fed normal-protein level diets (standard terminal ileal digestibility of 17.5%) containing CAS (N.CAS), CGM (N.CGM), or mixture of both of these two diets (CAS + CGM; N.MIX); and those fed low-protein diets (standard terminal ileal digestibility of 14.9%) containing CAS (L.CAS), CGM (L.CGM), or mixture of both of these two diets (CAS + CGM; L.MIX). According to our previous results [2], CAS and MIX diets result in more balanced release of AA during hydrolysis in vitro than the CGM diet.

**Table 1:** Ingredients and calculated nutrient compositions of the experimental diets (as-feed basis)

Ingredient (%)	Diet					
	Normal-protein diet			Low-protein diet		
	N.CAS	N.MIX	N.CGM	L.CAS	L.MIX	L.CGM
Corn	64.02	58.03	52.05	68.47	63.18	57.90
Wheat bran	2.00	2.00	2.00	2.00	2.00	2.00
Soybean meal	17.40	18.38	19.35	12.28	14.28	16.27
Casein	7.07	3.54	-	6.00	3.00	-
Corn gluten meal	-	7.34	14.68	-	5.27	10.54
Sucrose	3.00	3.00	3.00	3.00	3.00	3.00
Soybean oil	1.10	2.16	3.21	1.17	2.13	3.10
L-Arg	0.06	0.03	-	0.20	0.18	0.16
L-Ile	0.09	0.05	-	0.22	0.19	0.16
L-Leu	0.79	0.40	-	1.00	0.72	0.44
L-Cys	-	0.03	0.06	0.09	0.11	0.13
L-Tyr	-	0.21	0.41	0.24	0.300	0.36
L-His	-	0.02	0.04	0.07	0.10	0.12
L-Lys	0.28	0.45	0.61	0.48	0.60	0.73
L-Met	0.014	0.03	0.05	0.07	0.09	0.11
L-Phe	-	0.23	0.45	0.26	0.33	0.39
L-Thr	0.12	0.12	0.11	0.22	0.22	0.22
L-Trp	0.007	0.02	0.04	0.04	0.05	0.06
L-Val	0.05	0.04	0.03	0.16	0.16	0.17
Limestone	1.00	1.01	1.02	0.88	0.99	1.09
Dicalcium phosphate	1.15	1.10	1.05	1.18	1.18	1.19
Salt	0.85	0.84	0.84	0.85	0.85	0.84
Premix	1.00	1.00	1.00	1.00	1.00	1.00
Total	100.00	100	100.00	100.00	100	100.00
Nutritional levels, %						
Net energy, MJ/kg	10.26	10.255	10.25	10.25	10.26	10.26
Crude fiber	1.73	1.74	1.75	1.63	1.67	1.70
Ca	0.80	0.8	0.80	0.80	0.8	0.80
Available P	0.40	0.4	0.40	0.40	0.4	0.40
SID						
Crude protein	17.54	17.54	17.53	14.89	14.89	14.88
Arg	1.06	1.06	1.06	1.07	1.07	1.07
His	0.49	0.49	0.49	0.48	0.48	0.48
Ile	0.85	0.85	0.85	0.85	0.85	0.85
leu	2.43	2.425	2.42	2.43	2.43	2.43
Lys	1.35	1.35	1.35	1.35	1.35	1.35
Met + Cys	1.24	1.24	1.24	1.24	1.24	1.24
Phe + Tyr	3.26	3.26	3.25	3.25	3.25	3.25
Thr	0.79	0.79	0.79	0.79	0.79	0.79
Trp	0.22	0.22	0.22	0.22	0.22	0.22
Val	0.94	0.925	0.91	0.86	0.86	0.86

Premix providing per kg of complete diet: vitamin A, 28,500 IU; vitamin D, 36,000 IU; vitamin E, 67.5 IU; vitamin K, 37.5 mg; vitamin B, 17.5 mg; vitamin B_1_, 0.075 mg; nicotinamide, 70 mg; folic acid, 3 mg; D-calpanate, 37.5 mg; D-biotin, 0.375 mg; antioxidant, 0.15 mg; choline chloride, 105 mg; Co, 1 mg; Cu, 155; Fe, 145 mg; Mn, 75 mg; Zn, 125 mg; I, 0.3 mg; Se, 0.3 mg

### Experimental protocol and sample collection

After 5 days of adaptation to their environment, the weaned pigs were fed the experimental diets for 28 days. The piglets were fed three times a day and had free access to water. On day 28, after feeding for 2 h, the piglets were anesthetized, blood samples were obtained, and the samples were centrifuged for 20 min at 2,000 rpm/min [14]. The supernatants were collected and stored at -80℃ for measurement of D-lactic acid and diamine oxidase (DAO) concentrations. The first third of the jejunum, ileum, and cecum were sampled at a location of approximately 3 cm. Samples of digesta were collected from the jejunum, ileum, and cecum of each animal. Digesta samples were snap-frozen in liquid nitrogen and stored at − 80℃ until use (see Chang et al. 2018 [15] for more details about the methods used).

### Measurement of serum D-lactic acid and DAO concentrations by ELISA

The serum D-lactic acid and DAO concentrations were determined using Swine ELISA kits (Enzyme label, China).The absorbance of each well was measured at 450 nm using a microplate reader (BioTek Instruments, Gene Company Limited, USA), according to the manufacturer’s instructions.

### 16S gene sequencing of the gut microbiota and analysis of its biodiversity

The microbial DNA in samples of digesta collected from the jejunum, ileum, and cecum was extracted using a kit from MP Biomedicals (Santa Ana, CA, USA), and the quality and quantity of the extracted DNA was assessed using 0.8% agarose gel electrophoresis and a NanoDrop ND-1000 spectrophotometer (Thermo Fisher Scientific, Waltham, MA, USA), respectively. The V3–V4 region of the 16S rRNA was amplified using a previously reported PCR method [16], and the primer sequences were 338F 5’-ACTCCTACGGGAGGCAGCA-3’ and 806R 5’-GGACTACHVGGGTWTCTAAT-3’. The resulting amplicons were sequenced using the Illumina MiSeq platform and a MiSeq Reagent Kit v3 at Shanghai Personal Biotechnology, Co., Ltd. (Shanghai, China).

The sequences generated were analyzed using Quantitative Insights into Microbial Ecology, v1.8.0 (QIIME) [17] for taxonomic classification at the phylum and genus levels. Mothur [18] was used to measure the following indices of alpha diversity: Shannon index, ACE index, Chao1 index, and Simpson index.

### Statistical analysis

Data are presented as mean ± standard error and analyzed using a univariate general linear model in SPSS software v20.0 (IBM, Inc., Armonk, NY, USA), with PL denoting differences in the dietary protein content, PS denoting differences in the protein source, and PL × PS denoting an interaction. When there was an interaction between protein content and protein source, the six diets were compared using Duncan’s multiple comparison tests. *P* < 0.05 was considered to represent statistical significance.

## Results

### Serum concentrations of D-lactic acid and DAO

To study the effects of dietary protein sources and content on the intestinal permeability of weaned pigs, we first measured the serum concentrations of D-lactic acid and DAO, and the results are shown in Table 2. The DAO concentration tended to be higher in pigs fed a low protein diet than in those fed a normal protein diet (0.05 < *P* < 0.1). The DAO concentration in the CGM groups was significantly higher than those in the CAS and MIX groups (*P* < 0.05).

**Table 2:** Serum concentrations of DAO and D-lactic acid

	PL		PS			NP			LP			SEM	*P*		
	NOR	LOW	CAS	CGM	MIX	CAS	CGM	MIX	CAS	CGM	MIX		PL	PS	PL × PS
DAO (ng/mL)	332.67	369.11	325.74^x^	401.19^y^	325.76^x^	319.23	380.33	298.45	332.24	422.05	353.06	9.608	0.069	0.040	0.649
D-D-D-lactic acid (μmol/mL)	662.27	710.86	671.84^xy^	746.25^x^	641.61^y^	650.98	732.50	603.34	692.70	760.00	679.89	16.901	0.163	0.050	0.840

*PL* protein levels, *PS* protein sources, *NP* normal-protein level, *LP* low-protein level, *NOR* normal-protein level group; x, y, and z: protein source differences (*P* < 0.05)

### Composition and diversity of the gut microbiota

We next compared the microbial diversity of the intestinal contents of the groups, and the results are shown in Table 3. The Chao1 and ACE indices for the jejunum of the low-protein groups were significantly higher than those for the normal-protein groups (*P* < 0.05), and the Simpson and Shannon indices of the CGM groups were significantly higher than those of the CAS and MIX groups (*P* < 0.05). The Shannon indices for the ileum of the low-protein groups were significantly lower than those for the normal-protein groups (*P* < 0.05). There was no significant differences in the diversity of intestinal microbiota in the cecum (*P* > 0.05).

**Table 3:** Diversity of the microbiota in the intestinal content samples, determined using sequencing of the 16S rRNA genes

	PL		PS			NP			LP			SEM	*P*		
	NOR	LOW	CAS	CGM	MIX	CAS	CGM	MIX	CAS	CGM	MIX		L	S	L × S
Jejunum															
Simpson	0.891	0.840	0.827^y^	0.931^x^	0.840^y^	0.8950	0.9418	0.8356	0.7581	0.9195	0.8434	0.014	0.079	0.011	0.099
Shannon	4.827	5.097	4.360^y^	5.798^x^	4.728^y^	4.678	5.986	4.628	4.043	5.610	4.828	0.120	0.269	< 0.001	0.347
Chao1	863.69 ^n^	964.58 ^m^	874.56	915.10	952.75	818.28	817.22	955.57	930.84	1012.97	949.94	21.517	0.028	0.366	0.153
Ace	893.12 ^n^	988.10 ^m^	899.43	934.61	987.80	836.87	836.11	1006.39	961.99	1033.11	969.19	21.464	0.038	0.267	0.078
Ileum															
Simpson	0.843	0.801	0.835	0.817	0.814	0.7967^abc^	0.8881^a^	0.8432^ab^	0.8732^abc^	0.7450^c^	0.7855^b^	0.012	0.105	0.764	0.005
Shannon	4.761 ^m^	4.231 ^n^	4.406	4.501	4.581	4.180^bc^	5.272^a^	4.832^ab^	4.632^ab^	3.730^c^	4.330^bc^	0.101	0.016	0.778	0.002
Chao1	857.03	859.65	851.08	829.34	894.60	818.51^b^	756.24^b^	996.36^a^	883.65^b^	902.44^ab^	792.85^b^	23.907	0.957	0.546	0.019
Ace	868.57	894.30	856.53	872.74	915.04	824.00^bc^	778.70^c^	1003.03^a^	889.07^abc^	966.79^ab^	827.06^bc^	22.506	0.574	0.546	0.012
Cecum															
Simpson	0.927	0.914	0.910	0.946	0.9062	0.8906	0.9616	0.9301	0.9288	0.9305	0.8829	0.008	0.406	0.102	0.079
Shannon	6.349	6.177	6.092	6.582	6.115	5.846	6.832	6.370	6.338	6.332	5.860	0.100	0.396	0.102	0.076
Chao1	1210.19	1159.85	1163.04	1160.62	1231.40	1129.13	1274.10	1227.36	1196.95	1047.15	1235.44	28.084	0.380	0.502	0.114
Ace	1227.36	1169.21	1174.13	1176.00	1244.73	1149.44	1285.99	1246.65	1198.82	1066.01	1242.81	26.570	0.286	0.462	0.130

*PL* protein levels, *PS* protein sources, *NP* normal-protein level, *LP* low-protein level, *NOR* normal-protein group; m, n: protein levels; x, y, and z: protein source differences; a, b, and c: differences between the N.CAS, N.CGM, N.MIX, L.CAS, L.CGM, and L.MIX groups (*P* < 0.05)

**Fig. 1:**
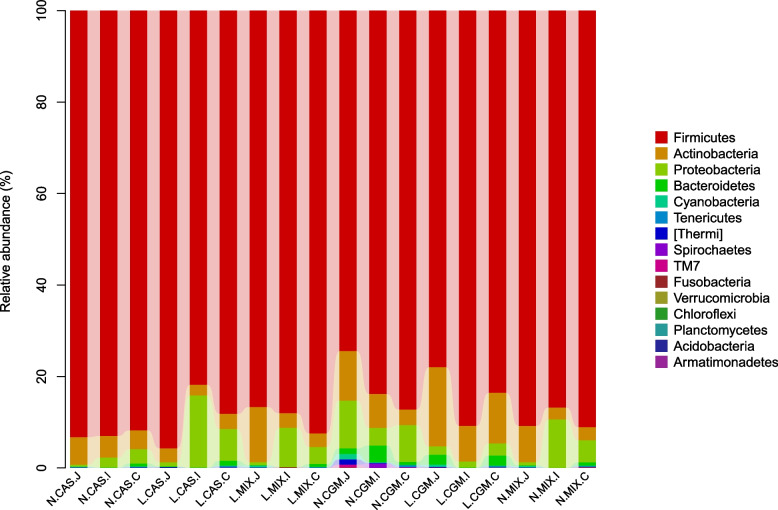
Composition of the microbiota in each intestinal segment at the phylum level. J, jejunum; I, ileum; C, cecum

*Firmicutes* were the most dominant phylum in all the intestinal segments, followed by *Proteobacteria*, *Actinobacteria*, *Cyanobacteria*, and *Bacteroidetes* (Fig. 1, Supplementary Table 1). There were no protein content-related differences in the abundances of these phyla in the ileum or cecum, but low-protein diets significantly reduced the abundance of *Proteobacteria* in the jejunum (*P* < 0.01), and the abundances of the main phyla with the exception of the *Firmicutes* phylum in the jejunum were higher in the CGM group than in the CAS and MIX groups (*P* < 0.05). In the jejunum, the abundance of *Firmicutes* in the N.CGM group was significantly lower than in the N.CAS, N.MIX, L.CAS, and L.MIX groups (*P* < 0.05).

**Fig. 2:**
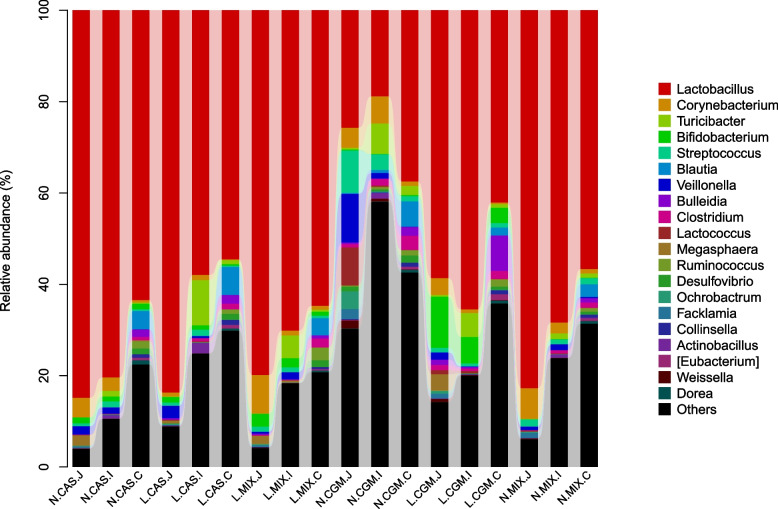
Compositions of the microbiota in each intestinal segment at the genus level. J, jejunum; I, ileum; C, cecum

At the genus level, *Lactobacillus* was the most dominant genus in all the intestinal segments (Fig. 2). *Lactobacillus* was significantly more abundant in the jejunum of piglets fed a low-protein diet than in those fed a normal-protein diet (*P* < 0.05), and *Bifidobacterium* was more abundant in the CAS and MIX groups than in the CGM group in the jejunum (*P* < 0.05). Finally, *Lactobacillus* and *Bifidobacterium* were have more abundant in the jejunum and ileum in the L.MIX groups than in the other groups. There were not significant differences in intestinal microbiota diversity in the cecum. At the family level, the abundance of *Enterobacteriaceae* in the ileum was significantly lower in the low-protein diets groups than in the fed a normal-protein diet groups (*P* < 0.05). *Lactobacillus* was significantly more abundant in each intestineal segment of the CAS and MIX groups than in the CGM groups (*P* < 0.05). In addition, the *Clostridiaceae* were more abundant in the CGM groups than in the CAS and MIX groups (*P* < 0.05) in each intestinal segment (Supplementary Table 2).

According to the results of amino acid release dynamics in Hu et al. 2022, according to the results of amino acid release dynamics in Hu et al. 2022 [2], the larger the area between individual FAA curve and TFAA curve, the more asynchronous the amino acid release dynamics are. Then we analyzed the correlation between amino acid release dynamics and intestinal microbial diversity at the phylum level and genus level. The results showed that Amino acid release asynchronism (AARA) was a significant negative correlation with *Firmicutes* in the jejunum (Table 4), but there was no significant correlation with microbial diversity at phylum level in cecum. Amino acid release asynchronism (AARA) was a significant positive correlation with *Clostridiaceae* in jejunum and ileum, and that was a significant negative correlation with *Lactobacillus* in the jejunum (Table 5), and there was no significant correlation with microbial diversity at genus level in cecum. The diet with more balanced amino acid release dynamics was more conducive to the accumulation of beneficial bacteria and inhibited the proliferation of harmful bacteria in the small intestine, but had little effect on the microbes in the cecum.

**Table 4:** Correlation analysis between amino acid release dynamics and intestinal microbial diversity at the phylum level

		Jejunum					Ileum					Cecum				
		*Firmicutes*	*Actinobacteria*	*Proteobacteria*	*Cyanobacteria*	*Bacteroidetes*	*Firmicutes*	*Actinobacteria*	*Proteobacteria*	*Cyanobacteria*	*Bacteroidetes*	*Firmicutes*	*Actinobacteria*	*Proteobacteria*	*Cyanobacteria*	*Bacteroidetes*
AARA	R2	-.934^**^	-.436	.888^*^	.986^**^	.579	-.370	-.093	.049	.872^*^	.662	-.465	-.427	.376	.131	-.069
	*P*	.029	.573	.002	.004	.201	.197	.779	.895	.016	.030	.230	.565	.374	.998	.437

*AARA* Amino acid release asynchronism. According to the results of amino acid release dynamics in Hu et al. 2022 [2], the larger the area between individual FAA curve and TFAA curve, the more asynchronous the amino acid release dynamics are. **P < 0.01, *P < 0.05

**Table 5:** Correlation analysis between amino acid release dynamics and intestinal microbial diversity at the genus level

		Jejunum					Ileum					Cecum				
		*Lactobacillus*	*Clostridiaceae*	*Enterobacteriaceae*	*Bifidobacterium*	*Corynebacterium*	*Lactobacillus*	*Clostridiaceae*	*Enterobacteriaceae*	*Bifidobacterium*	*Corynebacterium*	*Lactobacillus*	*Clostridiaceae*	*Enterobacteriaceae*	*Bifidobacterium*	*Corynebacterium*
AARA	R^2^	-.923^**^	.821^*^	.878^*^	-.598	.104	-.004	.935^**^	-.097	-.477	.068	-.777	.791	.062	-.359	.468
	*P*	.000	.011	.001	.064	.878	.664	.000	.949	.086	.657	.015	.029	.734	.423	.501

*AARA* Amino acid release asynchronism. According to the results of amino acid release dynamics in Hu et al. 2022 [2], the larger the area between individual FAA curve and TFAA curve, the more asynchronous the amino acid release dynamics are. **P < 0.01, *P < 0.05

## Discussion

The piglet is a well-studied model for investigations into intestinal health, given their special digestive physiology. Furthermore, diarrhoea, disease or even death is more commonly observed in this model because piglets have an imperfect and impaired gastrointestinal tract. An impaired intestinal barrier is associated with increased permeability, which can facilitate the flow of intestinal bacteria and toxins into the body, affecting animal health [19, 20].

The serum concentrations of DAO and D-lactic acid are used as indicators of intestinal permeability, thus, they can be used to appraise epithelial barrier function [21]. In the present study, we showed that the serum concentrations of DAO and D-lactic acid are higher in piglets fed a low-protein diet, which may at least in part explain the poor growth performance of piglets on such diets as reported in previous studies [16]. However, this was not significant, which may be because supplementation using monomeric amino acids may satisfy the requirements for normal growth and the development of the intestinal mucosal barrier. We found that diets containing CAS and MIX promote intestinal mucosal barrier function, and we previously showed that they are superior to CGM for growth and nitrogen deposition in weaned pigs, which may be because of the more balanced release of AA from CAS and MIX diets [2]. This necessitates greater supplementation of the diet with monomeric amino acids, such that the amino acid release of amino acids from the diet is not synchronized, which may be associated with damage to the intestinal mucosal barrier system.

The gastrointestinal tract (GIT) of piglets contains a complex and dynamic microbial ecosystem that plays an important role in nutrient digestion, absorption and metabolism, as well as maintenance of intestinal health [22–24]. Gut microbes play a key role in the maintenance of intestinal health [25], and changes in the composition of the gut microbiota may affect protein metabolism and microbial metabolite generation [26]. For example, lowering the protein content of the diet of growing pigs has been shown to limit the amount of protein available for protein-fermenting bacteria [27]. Furthermore, an increase in dietary protein content is associated with reductions in the number of beneficial bacteria, such as *Lactobacillus* [28] and *Bifidobacterium* [29], and increases in the number of harmful bacteria, such as Escherichia coli, resulting in intestinal injury and diarrhoea. By contrast, weaned pigs consuming a low-protein diet with a balanced amino acid composition have fewer E. coli in their guts [30], which is associated with fewer symptoms of diarrhoea [31]. Similarly, in the present study, we found that a low-protein diet reduced the abundance of *Proteobacteria*, and increased the abundance of *Lactobacillus* in the jejunum. Furthermore, the *Enterobacteria* were less abundant in the ileum of the low-protein group than in those of the normal-protein group.

By virtue of their different structures and amino acid release characteristics, different protein sources have contrasting effects on gut microbes. Here, we found that in the jejunum the CGM group had a significantly more diverse population according to the Simpson and Shannon indexes and various phyla were more abundant in this group than in those of the CAS and MIX groups. Furthermore, compared with the CAS and MIX groups, *Bacteroidetes* and *Cyanobacteria* in the CGM group were significantly more abundant and the number of members of the *Clostridiaceae* and *Enterobacteriaceae* were significantly higher in the jejunum. This is similar to the findings of a previous study, which showed that the slower the release rate of protein, the more easily the protein is digested and absorbed by harmful bacteria, such as E. coli, causing their proliferation [32]. CAS can be digested by host enzymes in the proximal small intestine, thereby reducing its degradation by E. coli. Instead, a CAS-based diet is associated with greater diversity of *Lactobacillus* and *Bifidobacterium* [33], and reductions in the number of fecal *Staphylococcus*, E. coli, and *Streptococcus* [34]. Similarly, in the present study, the CAS and MIX groups had significantly more abundant beneficial bacteria, such as *Lactobacillus* and *Bifidobacterium*, and fewer harmful bacteria in the jejunum. Our results show that the protein content is no significant differences in intestinal tight junction protein expression in the ileum and cecum, which might be a possible reason behind this. And there was the same in the previous study [16]. In addition, the microbial ecosystems in each pig’s gut continue to change as pigs grow, and the variation of the gut bacterial populations of swine is caused by a variety of factors [35]. Our results show that the release kinetics of amino acids can change the number of some bacteria species in the cecum of piglets, without increasing diversity. According to the results of Knapp et al. (2022) [36], there is no significant difference in faecal flora between the ceca of excised and unexcised piglets fed the same diet.

## Conclusion

A reduction in dietary protein content of ~ 3% and supplementation with the appropriate amount of amino acids does not affect the intestinal barrier function of piglets, and increases the number of beneficial bacteria in their intestines. In addition, the balanced release of amino acids that characterizes the CAS and MIX diets helps to maintain intestinal morphology, reduce intestinal permeability, and maintain intestinal barrier function. Furthermore, it increases the number of beneficial bacteria, such as *Lactobacillus* and *Bifidobacterium*, in the intestine, and optimizes the intestinal microecology, which is also beneficial for intestinal health. These findings provide information that should facilitate the improvement of the performance of weaned pigs and the development of new environment-friendly diets.

## Supplementary Information

**Additional file 1.**

## Data Availability

The datasets analyzed during the current study are available from the corresponding author on reasonable request.
