# Supplementary material for: Effects of diets with different amino acid release characteristics on the gut microbiota and barrier function of weaned pigs
Source: BMC Microbiol. 2023 Jan 19;23:18. doi: 10.1186/s12866-023-02762-8 (PMC9850806; doi:10.1186/s12866-023-02762-8)
Supplement: Supplementary file 1 — Additional file 1. [file 12866_2023_2762_MOESM1_ESM.docx]

Table 1. Gut microbial composition in each intestinal segment at the phylum level

|  | PL | | PS | | | NP | | | LP | | | SEM | *P* | | |
| --- | --- | --- | --- | --- | --- | --- | --- | --- | --- | --- | --- | --- | --- | --- | --- |
|  | NOR | LOW | CAS | CGM | MIX | CAS | CGM | MIX | CAS | CGM | MIX |  | L | S | L×S |
| Jejunum | | | | | | | | | | | | | | | |
| *Firmicutes* (%) | 86.160 | 86.753 | 94.480^x^ | 76.180^y^ | 88.710^x^ | 93.26 | 74.42 | 90.80 | 95.70 | 77.94 | 86.62 | 1.225 | 0.811 | <0.001 | 0.395 |
| *Actinobacteria* (%) | 8.240 | 10.592 | 4.490^z^ | 15.648^x^ | 8.110^y^ | 5.98^cd^ | 10.82^b^ | 7.92^bd^ | 3.00^c^ | 20.48^a^ | 8.30^bd^ | 0.704 | 0.109 | <0.001 | <0.001 |
| *Proteobacteria* (%) | 3.873 ^m^ | 1.127 ^n^ | 0.680^y^ | 6.130^x^ | 0.690^y^ | 0.42^b^ | 10.46^a^ | 0.74^a^ | 0.94^a^ | 1.80^a^ | 0.64^a^ | 0.469 | <0.001 | <0.001 | <0.001 |
| *Bacteroidetes* (%) | 0.533 | 0.780 | 0.130^y^ | 1.650^x^ | 0.190^y^ | 0.16 | 1.14 | 0.30 | 0.10 | 2.16 | 0.05 | 0.229 | 0.595 | <0.001 | 0.495 |
| *Cyanobacteria* (%) | 0.460 | 0.320 | 0.060^y^ | 0.850^x^ | 0.260^y^ | 0.06 | 1.20 | 0.12 | 0.06 | 0.50 | 0.40 | 0.099 | 0.487 | <0.001 | 0.138 |
| Ileum | | | | | | | | | | | | | | | |
| *Firmicutes* (%) | 87.860 | 90.912 | 89.600 | 87.300 | 91.258 | 93.00 | 83.84 | 86.74 | 86.20 | 90.76 | 95.78 | 1.513 | 0.324 | 0.562 | 0.095 |
| *Actinobacteria* (%) | 3.702 | 2.261 | 3.497 | 2.590 | 2.858 | 2.16 | 3.88 | 5.07 | 4.83 | 1.30 | 0.65 | 0.589 | 0.236 | 0.806 | 0.071 |
| *Proteobacteria* (%) | 3.532 | 3.588 | 3.530 | 4.250 | 2.900 | 4.72 | 3.28 | 2.60 | 2.34 | 5.23 | 3.20 | 0.511 | 0.956 | 0.578 | 0.231 |
| *Bacteroidetes* (%) | 0.036 | 0.028 | 0.033^xy^ | 0.051^x^ | 0.012^y^ | 0.03^ab^ | 0.06^a^ | 0.01b | 0.03^ab^ | 0.04^ab^ | 0.01^b^ | 0.005 | 0.448 | 0.018 | 0.545 |
| *Cyanobacteria* (%) | 0.018 | 0.007 | 0.008^y^ | 0.023^x^ | 0.006^y^ | 0.01^b^ | 0.04^a^ | 0.00b | 0.00^b^ | 0.01^b^ | 0.01^b^ | 0.003 | 0.059 | 0.025 | 0.012 |
| Cecum | | | | | | | | | | | | | | | |
| *Firmicutes* (%) | 89.953 | 88.047 | 89.870 | 85.380 | 91.750 | 91.58 | 87.20 | 91.08 | 88.16 | 83.56 | 92.42 | 1.397 | 0.501 | 0.182 | 0.716 |
| *Actinobacteria* (%) | 3.440 | 4.467 | 5.530 | 30.50 | 3.280 | 4.08 | 3.40 | 2.84 | 6.98 | 2.70 | 3.72 | 0.575 | 0.381 | 0.172 | 0.452 |
| *Proteobacteria* (%) | 4.097 | 3.743 | 3.250 | 4.600 | 3.910 | 3.20 | 4.25 | 4.84 | 3.30 | 4.95 | 2.98 | 0.397 | 0.661 | 0.410 | 0.400 |
| *Bacteroidetes* (%) | 0.680 | 1.300 | 0.860 | 0.620 | 1.490 | 0.64 | 0.64 | 0.76 | 1.08 | 0.60 | 2.22 | 0.321 | 0.345 | 0.530 | 0.629 |
| *Cyanobacteria* (%) | 0.026 | 0.026 | 0.024 | 0.031 | 0.024 | 0.04^ab^ | 0.03^ab^ | 0.01^b^ | 0.02^ab^ | 0.03^ab^ | 0.04^ab^ | 0.004 | 0.960 | 0.631 | 0.031 |

PL: protein levels; PS: protein sources; NP: normal-protein level; LP: low-protein level; NOR: normal-protein group; m, n: protein levels; x, y, and z: protein source differences; a, b, and c: differences between the N.CAS, N.CGM, N.MIX, L.CAS, L.CGM, and L.MIX groups (*P*<0.05).

Table 2. Composition of the microbiota in each intestinal segment at the genus level

|  | PL | | PS | | | NP | | | LP | | | SEM | *P* | | |
| --- | --- | --- | --- | --- | --- | --- | --- | --- | --- | --- | --- | --- | --- | --- | --- |
|  | NOR | LOW | CAS | CGM | MIX | CAS | CGM | MIX | CAS | CGM | MIX |  | L | S | L×S |
| Jejunum | | | | | | | | | | | | | | | |
| *Lactobacillus* (%) | 64.423^n^ | 75.748^m^ | 84.230^x^ | 42.145^y^ | 83.882^x^ | 84.82^a^ | 25.70^c^ | 82.75^a^ | 83.64^a^ | 58.59^b^ | 85.01^a^ | 1.958 | <0.001 | <0.001 | <0.001 |
| *Clostridiaceae* (%) | 1.905 | 0.859 | 0.519^y^ | 2.514^x^ | 1.112^y^ | 0.18 | 3.69 | 1.84 | 0.86 | 1.33 | 0.38 | 0.259 | 0.056 | 0.014 | 0.079 |
| *Enterobacteriaceae* (%) | 0.070 | 0.029 | 0.026^y^ | 0.094^x^ | 0.029^y^ | 0.03 | 0.14 | 0.04 | 0.02 | 0.05 | 0.02 | 0.012 | 0.100 | 0.049 | 0.292 |
| *Bifidobacterium* (%) | 0.961 | 1.478 | 1.526^x^ | 0.248^y^ | 1.885^x^ | 1.31 | 0.21 | 1.37 | 1.75 | 0.29 | 2.40 | 0.266 | 0.342 | 0.047 | 0.765 |
| *Corynebacterium* (%) | 4.706^m^ | 2.889^n^ | 2.733^y^ | 3.070^y^ | 5.588^x^ | 4.29 | 4.38 | 5.46 | 1.18 | 1.77 | 5.72 | 0.412 | 0.039 | 0.022 | 0.232 |
| Ileum | | | | | | | | | | | | | | | |
| *Lactobacillus* (%) | 61.598 | 64.985 | 74.172^x^ | 36.232^y^ | 79.471^x^ | 80.38 | 27.05 | 77.37 | 67.97 | 45.42 | 81.57 | 2.734 | 0.543 | <0.001 | 0.094 |
| *Clostridiaceae* (%) | 12.683 | 10.010 | 7.196^y^ | 19.695^x^ | 7.148^y^ | 7.00 | 24.59 | 6.46 | 7.40 | 14.80 | 7.84 | 1.827 | 0.473 | 0.017 | 0.421 |
| *Enterobacteriaceae* (%) | 2.360^m^ | 0.642^n^ | 0.865 | 1.163 | 2.475 | 1.22 | 1.48 | 4.39 | 0.52 | 0.85 | 0.56 | 0.396 | 0.043 | 0.266 | 0.216 |
| *Bifidobacterium* (%) | 1.483 | 0.897 | 1.022^xy^ | 0.780^y^ | 1.768^x^ | 1.09 | 0.09 | 1.51 | 0.95 | 1.47 | 2.03 | 0.356 | 0.419 | 0.506 | 0.694 |
| *Corynebacterium* (%) | 1.924 | 1.500 | 2.022 | 2.179 | 0.935 | 2.93 | 2.01 | 0.83 | 1.12 | 2.35 | 1.04 | 0.297 | 0.482 | 0.194 | 0.270 |
| Cecum | | | | | | | | | | | | | | | |
| *Lactobacillus* (%) | 52.515 | 53.747 | 58.965^x^ | 39.752^y^ | 60.676^x^ | 63.44 | 37.45 | 56.65 | 54.49 | 42.05 | 64.70 | 2.852 | 0.831 | 0.011 | 0.449 |
| *Clostridiaceae* (%) | 3.216 | 2.728 | 1.258^y^ | 5.970^x^ | 1.688^y^ | 1.18 | 5.98 | 2.48 | 1.33 | 5.96 | 0.90 | 0.594 | 0.686 | <0.001 | 0.812 |
| *Enterobacteriaceae* (%) | 0.514 | 0.491 | 0.247 | 0.322 | 0.939 | 0.12 | 0.45 | 0.97 | 0.37 | 0.19 | 0.91 | 0.145 | 0.939 | 0.121 | 0.774 |
| *Bifidobacterium* (%) | 1.122 | 0.933 | 0.772 | 0.928 | 1.382 | 1.21 | 0.27 | 1.88 | 0.33 | 1.58 | 0.88 | 0.366 | 0.799 | 0.780 | 0.362 |
| *Corynebacterium* (%) | 0.839 | 0.449 | 0.365 | 0.669 | 0.898 | 0.55 | 0.98 | 0.98 | 0.18 | 0.36 | 0.81 | 0.128 | 0.141 | 0.253 | 0.771 |

PL: protein levels; PS: protein sources; NP: normal-protein level; LP: low-protein level; NOR: normal-protein group; m, n: protein levels; x, y, and z: protein source differences; a, b, and c: differences between the N.CAS, N.CGM, N.MIX, L.CAS, L.CGM, and L.MIX groups (*P*<0.05).
